# New cave-dwelling armored spiders (Araneae, Tetrablemmidae) from Southwest China

**DOI:** 10.3897/zookeys.388.5735

**Published:** 2014-03-13

**Authors:** Yucheng Lin, Shuqiang Li

**Affiliations:** 1 Key Laboratory of Bio-resources and Eco-environment (Ministry of Education), College of Life Sciences, Sichuan University, Chengdu, Sichuan 610064, China; 2 Institute of Zoology, Chinese Academy of Sciences, Beijing 100101, China

**Keywords:** Taxonomy, haplogyne spiders, new species, diagnosis, distribution

## Abstract

A new genus and five new species belonging to the family Tetrablemmidae are described from caves in Southwest China, i.e., *Sinammaoxycera* **gen. & sp. n.**, *Singaporemma banxiaoensis* **sp. n.**, *Singaporemma wulongensis* **sp. n.**, *Tetrablemma ziyaoensis* **sp. n.** and*Tetrablemma menglaensis* **sp. n.** The following new combination is proposed: *Sinamma sanya* (Lin & Li, 2010), **comb. n.** ex. *Shearella* Lehtinen, 1981. The relationships of the *Sinamma* **gen. n.** with other genera are discussed. Diagnoses and illustrations for all new taxa are given.

## Introduction

Tetrablemmids are medium-sized (Pacullinae) to small (Tetrablemminae) haplogyne spiders, characterized by a complex pattern of abdominal scuta (Shear 1978; Lehtinen 1981; Jocqué and Dippenaar-Schoeman 2006). They are mainly distributed in the tropical and subtropical regions where they are found in leaf litter, soil, and in caves (Burger et al. 2010). This family has been revised by Lehtinen (1981). Burger et al. (2006) and Burger (2008) studied the functional morphology of the genitalia of the family Tetrablemmidae. A total of 144 species belonging to 30 genera of the family Tetrablemmidae have been described (Platnick 2014).

Chinese tetrablemmids have been studied recently. Five species belonging to four genera from Hainan Province were reported by Tong and Li (2008). Lian (2009) reported one species of the genus *Perania* Thorell, 1890 from Yunnan Province. Lin and Li (2010) described five more species (two from rainforest habitats in Hainan and three from caves in the Yunnan-Guizhou Plateau). In total, 11 species belonging to eight genera have been reported from China (cf. Platnick 2014).

In the period of October 2010 to August 2011 we found several new and interesting species of the family Tetrablemmidae from Southwest China and in this paper we now describe five new tetrablemmid taxa.

## Material and methods

Specimens were examined and measured under an Olympus SZX7 stereomicroscope. Further details were studied under an Olympus BX43 compound microscope. All drawings were made using a drawing tube attached to an Olympus BX43 compound microscope, and then hand inked on ink jet plotter paper. Photographs were taken with a Canon EOS 60D wide zoom digital camera (8.5 megapixels). The images were combined using Helicon Focus 3.10 software. Male palp and female genitalia were examined and illustrated after they were dissected and detached from the spiders’ bodies. Vulvae were removed and treated in KOH solution before examination and illustration. To reveal the course of the sperm duct, the bulbs were treated in lactic acid and mounted in Hoyer’s Solution before examination and illustration. Left palp is illustrated unless missing, in which case the right palp is illustrated.

All measurements are provided in millimeters. Height of carapace is measured with tubercle. Leg measurements are given as total length (femur, patella, tibia, metatarsus, tarsus). The terminology mostly follows Lehtinen (1981) and Burger (2008). The abbreviations used in figures as follows: CP – central process; EP – epigynal pit; EF – epigynal fold; IVP – inner vulval plate; LH – lateral horn; PA – preanal scutum; PEG – perigenital scutum; POG – postepigastral scutum; SR – seminal receptaculum; VD – vulval duct; VS – vulval stem.

All specimens are acquired from caves by manual collection and preserved in 85% ethanol solution. All specimens are deposited in the Sichuan University Museum (SCUM) in Chengdu and in the Institute of Zoology, Chinese Academy of Sciences (IZCAS) in Beijing.

## Taxonomy

### Sinamma

Lin & Li gen. n.

http://zoobank.org/01376E2E-D204-4B55-8597-BD18F5AF0223

http://species-id.net/wiki/Sinamma

#### Type species.

*Sinamma oxycera* sp. n. from cave of Guangxi, China.

#### Etymology.

The generic name derives from the Latin word “*sina*” and “-*mma*” as a suffix of the genus *Tetrablemma*. The gender is feminine, with *sina* meaning China.

#### Diagnosis.

**Figure 1.**
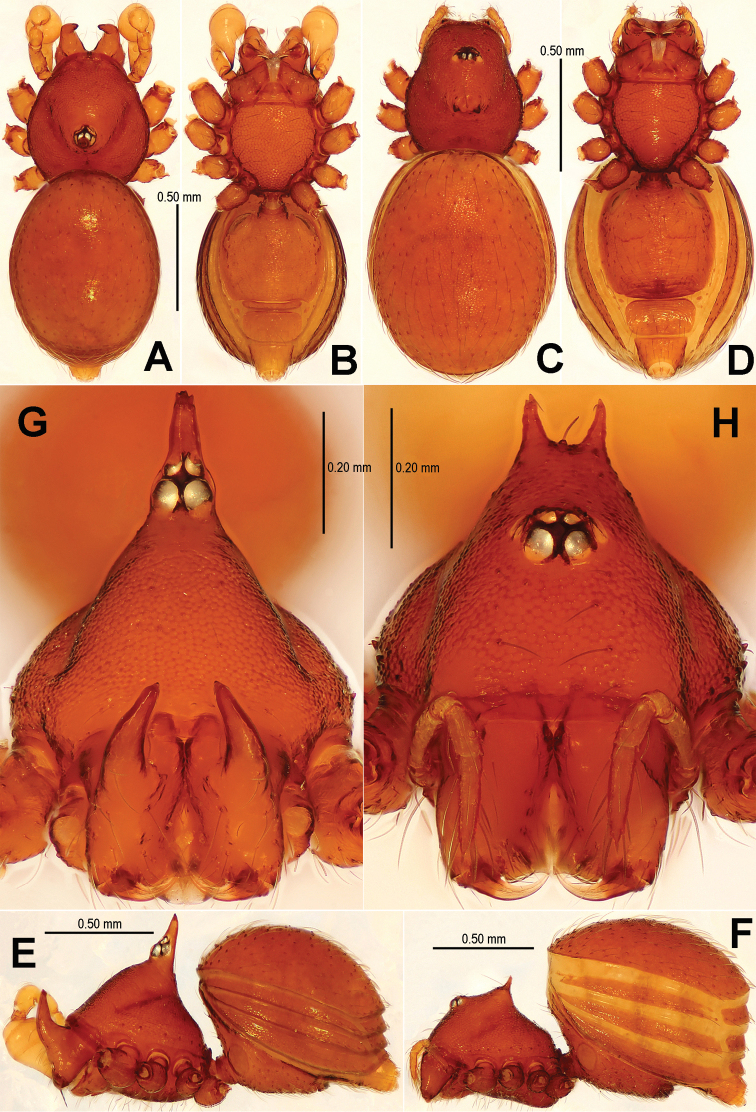
*Sinamma oxycera* gen. n. & sp. n., male holotype (**A–B, E, G**) and female paratype (**C–D, F, H**). **A–F** Habitus **G, H** Prosoma. **A, C** dorsal view **B, D** ventral view **E, F** lateral view **G, H** anterior view.

**Figure 2.**
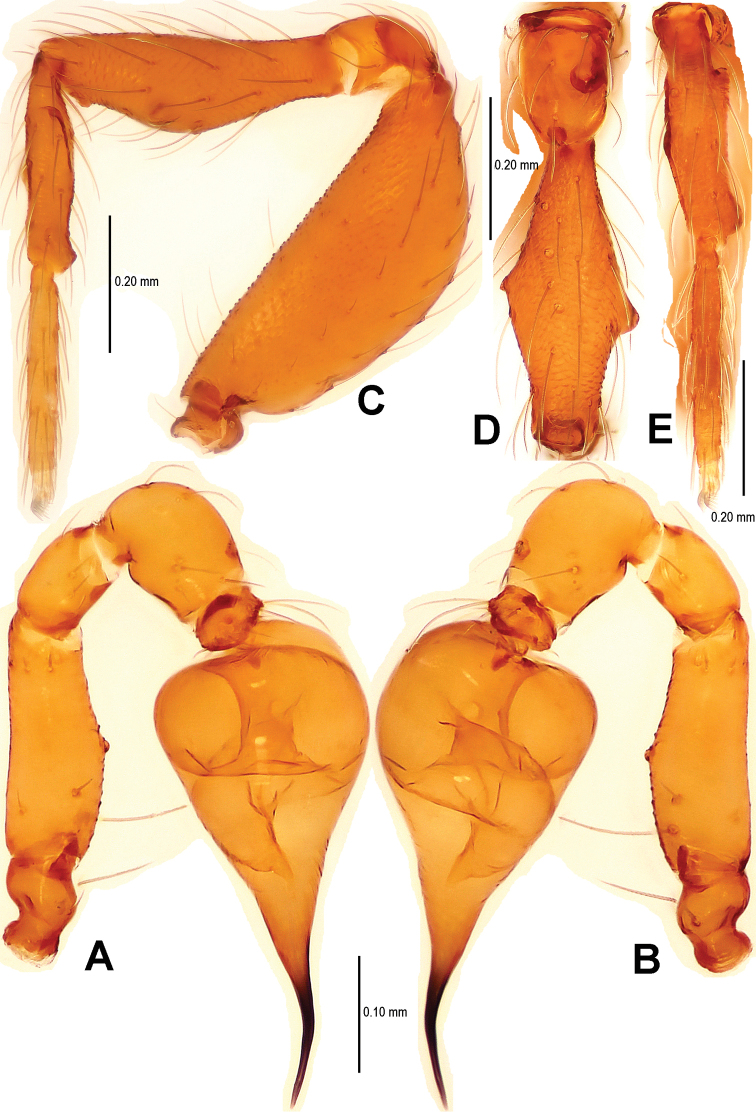
*Sinamma oxycera* gen. n. & sp. n., male holotype. **A, B** Left palp **C** Left leg I **D** Left tibia I **E** Left metatarsus I and tarsus I. **A** prolateral view **B, C** retrolateral view **D, E** anterior view.

**Figure 3.**
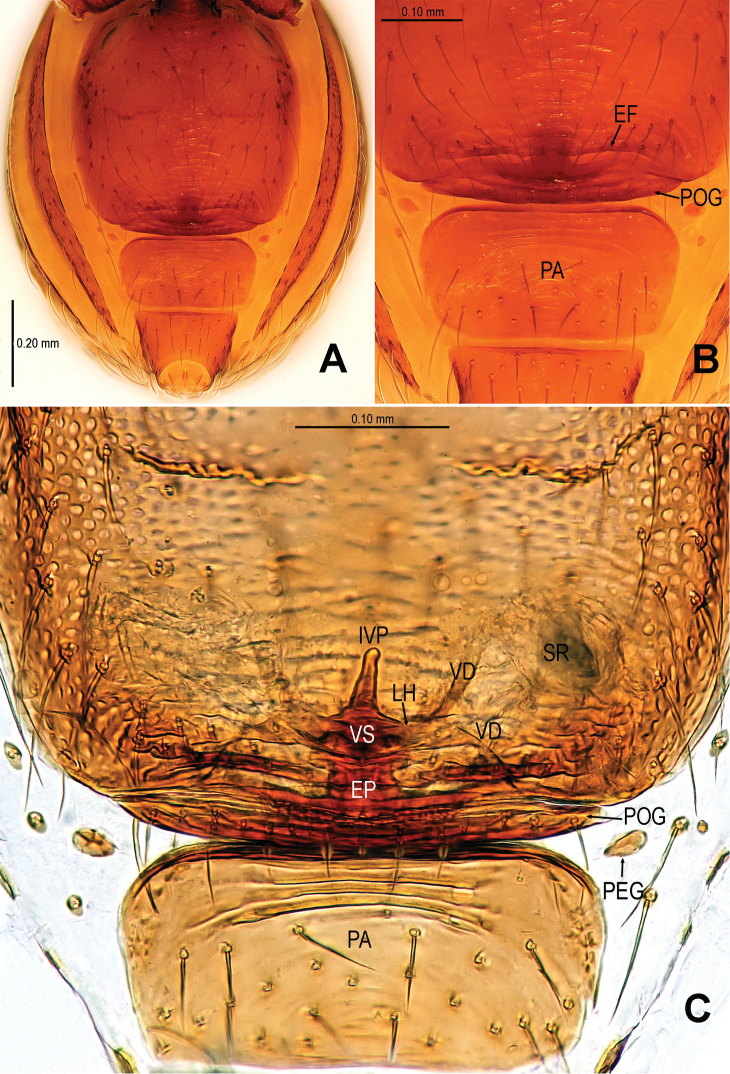
*Sinamma oxycera* gen. n. & sp. n., female paratype. **A** Opisthosoma **B** Genital area (untreated) **C** Cleared vulva (KOH-treated). **A, B** ventral view **C** dorsal view. Abbrs.: **EF** epigynal fold; **EP** epigynal pit; **IVP** inner vulval plate; **LH** lateral horn; **PA** preanal plate; **POG** postgenital plate; **SR** seminal receptaculum; **VD** vulval duct; **VS** vulval stem.

*Sinamma* gen. n. differs from all known genera of Tetrablemminae by the presence of a tubercle on the male carapace (Figs 1G, 1E; Lin and Li 2010: 24, figs 19, 20) and sometimes in females (Figs 1H, 1F; Lin and Li 2010: 25, figs 25, 26), the strongly modified male leg I (Figs 2C–E; Lin and Li 2010: 24, fig. 22), and by the exceptionally narrow postepigastral scutum in the both sexes (Figs 1B, 1D, 3A–C, 19B; Lin and Li 2010: 25, figs 27–28).

#### Description.

Small (1.2–1.6), six eyes compact in a group, male carapace usually with cephalic tubercle (present or absent in female). Male leg I robust, with tubercles at tibia and metatarsus. Cheliceral horn present (absent in female), much longer than in *Brignoliella* Shear, 1978 or *Shearella* Lehtinen, 1981, but shorter than in *Tetrablemma* O. P.-Cambridge, 1873 and *Gunasekara* Lehtinen, 1981. Abdomen oval, lateral scuta II–IV wide in both sexes.

Bulb long pyriform, embolus simple, needle-shaped; epigynal fold narrow; postepigastral scutum exceptionally narrow; central process absent, inner vulval plate well developed, vulval stem transverse.

#### Remarks.

The new genus *Sinamma* gen. n. contains two species and belongs to the subfamily Tetrablemminae. *Sinamma* gen. n. is similar to *Shearella* by the conical cheliceral horn and the pyriform bulb in the males, and is also similar to *Gunasekara* in having strongly modified leg I in the males. However, it can be distinguished from both *Shearella* and *Gunasekara* by the carapace having cephalic tubercle in male (sometimes in female also, e.g. *Sinamma oxycera* sp. n. and *Shearella sanya* Lin & Li, 2010), the presence of a distinct inner vulval plate in the female, and by the exceptionally narrow postepigastral scutum. The only oriental genus, *Singalangia* Lehtinen, 1981, for which the male is not yet known, has entirely different ocular pattern and vulval structures compared with those of *Sinamma* gen. n. In summary, *Sinamma oxycera* sp. n. and *Shearella sanya* Lin & Li share the following synapomorphies: an obvious cephalic tubercle, a strongly modified leg I in the male, and an exceptionally narrow postepigastral scutum in the female. These features distinguish them from other tetrablemmids. The new genus *Sinamma* is therefore proposed to accommodate these two oriental species, *Sinamma oxycera* sp. n. and *Sinamma sanya* (Lin & Li, 2010), comb. n., previously considered in *Shearella*.

#### Composition.

*Sinamma oxycera* sp. n. and *Sinamma sanya* (Lin & Li, 2010).

#### Distribution.

China (Guangxi, Hainan).

### Sinamma oxycera sp. n.

http://zoobank.org/104113CB-D708-415B-BD6F-1BBE847F6AFA

http://species-id.net/wiki/Sinamma_oxycera

Figs 1 – 3 , 16A–B , 19B , 22

#### Material.

Holotype ♂ and paratypes 2♀ (IZCAS), CHINA, Guangxi: Chongzuo City, Longzhou County, Shanglong Town, Xinlian Village, Gengyitun, Longmolai Cave, 22°29.809'N, 106°54.103'E, elevation ca. 224 m, 24 July 2011, Xiaoxiao Wang leg.

#### Etymology.

The specific name derives from the Greek word “*oxycerus*” = sharp horn, and refers to the sharp cephalic tubercle in the male; noun.

#### Diagnosis.

Males of *Sinamma oxycera* sp. n. can be distinguished from *Sinamma sanya* (see Lin and Li 2010: 23, figs 19–28) by a long cephalic tubercle (Figs 1G, E), the strongly modified leg I (Figs 2C–E), and the long pyriform palpal bulb (Figs 2A–B). Females can be recognized by a pair of cephalic tubercles (Fig. 1H), a wide, translucent vulval dorsal plate (Fig. 19B), a straight, long, inner vulval plate (Figs 3C, 19B), and the anteriorly wrinkled preanal scutum (Figs 3B, 19B).

#### Description.

**Male** (holotype). Coloration: body reddish-brown; legs yellowish-brown. Measurements: total length 1.48; carapace 0.68 long, 0.57 wide, 0.57 high; abdomen 0.95 long, 0.71 wide, 0.69 high; clypeus 0.48 high; sternum 0.41 long, 0.41 wide. Length of legs: I 1.89 (0.62, 0.20, 0.42, 0.33, 0.33); II 1.79 (0.57, 0.18, 0.41, 0.30, 0.32); III 1.61 (0.47, 0.16, 0.37, 0.29, 0.32); IV 2.15 (0.66, 0.18, 0.55, 0.40, 0.36).

Carapace (Figs 1A, E, G) finely reticulated, margin rugose; ocular area raised, cephalic tubercle long, sharp (Figs 1G, E); clypeus very high, anterior margin rounded (Figs 1A, G); clypeal area slightly convex; cheliceral horn long, basally wide, distally crooked (Figs 1G, E); sternum with sparse setae, margins rugose (Fig. 1B). Legs: femur I swollen (Fig. 2C); tibiae I–III with 3 trichobothria, tibia IV with 4 trichobothria, and metatarsi I-IV with a thichobothrium; tibia I medially wide, with two small lateral tubercles; metatarsus I with a proximal and a distal tubercle (Figs 2C–E).

Abdomen (Figs 1A–B, E) dorsal scutum oval, finely granulated; ventral scutum reticulated, margin striated; lateral scutum I short; postepigastral scutum exceptionally narrow, subequal in width to preanal scutum (Fig. 1B).

Palp (Figs 2A–B, 16A–B): femur slightly swollen, ventrally granulated; patella approximately 1/2 femur in length; tibia smooth, swollen; bulb long, pyriform, smooth; embolus long, curved slightly, strongly sclerotized; sperm duct extending, visible through the bulbal integument.

**Female** (paratype). Coloration: same as in male.

Measurements: total length 1.59; carapace 0.68 long, 0.53 wide, 0.41 high; abdomen 1.02 long, 0.84 wide, 0.88 high; clypeus 0.23 high; sternum 0.40 long, 0.39 wide. Length of legs: I 1.89 (0.61, 0.18, 0.46, 0.31, 0.34); II 1.73 (0.55, 0.16, 0.41, 0.29, 0.32); III 1.61 (0.46, 0.15, 0.39, 0.29, 0.31); IV 2.13 (0.64, 0.17, 0.55, 0.39, 0.37).

Carapace (Figs 1C, H and F) with a pair of cephalic tubercles; cephalic part slightly elevated, clypeus lower than in male; cheliceral horn absent. Legs as in male, except for leg I undecorated.

Abdomen (Figs 1C–D, F; 3A): lateral scutum I anteriorly extending beyond the anterior rim of operculum; preanal scutum anteriorly rugose, covered with sparse serrated setae.

Genitalia (Figs 3B–C; 19B): vulval stem transverse, sclerotized; lateral horns robust, supporting the base of vulval ducts; vulval duct narrow, weakly sclerotized; spermathecae translucent, rugose, membranous; vulval dorsal plate wide, fused to lateral horn and base of vulval ducts; inner vulval plate finger-shaped, sclerotized, basally wide; central process absent.

#### Distribution.

Known only from the type locality (Fig. 22).

### Singaporemma

Shear, 1978

http://species-id.net/wiki/Singaporemma

#### Type species.

*Singaporemma singularis* Shear, 1978 from Singapore.

#### Diagnosis.

Distinguished from other tetrablemmids by the swollen palpal tibia, the egg-shaped bulb, the originating position of embolus in male, and by the oval epigynal pit, the central process larger than inner vulval plate in female.

#### Distribution.

Four species are known from China, Singapore and Vietnam before the current study.

### Singaporemma banxiaoensis sp. n.

http://zoobank.org/EB434E0D-E8B9-4D2D-879A-C8C39DB4AF70

http://species-id.net/wiki/Singaporemma_banxiaoensis

**Figure 4.**
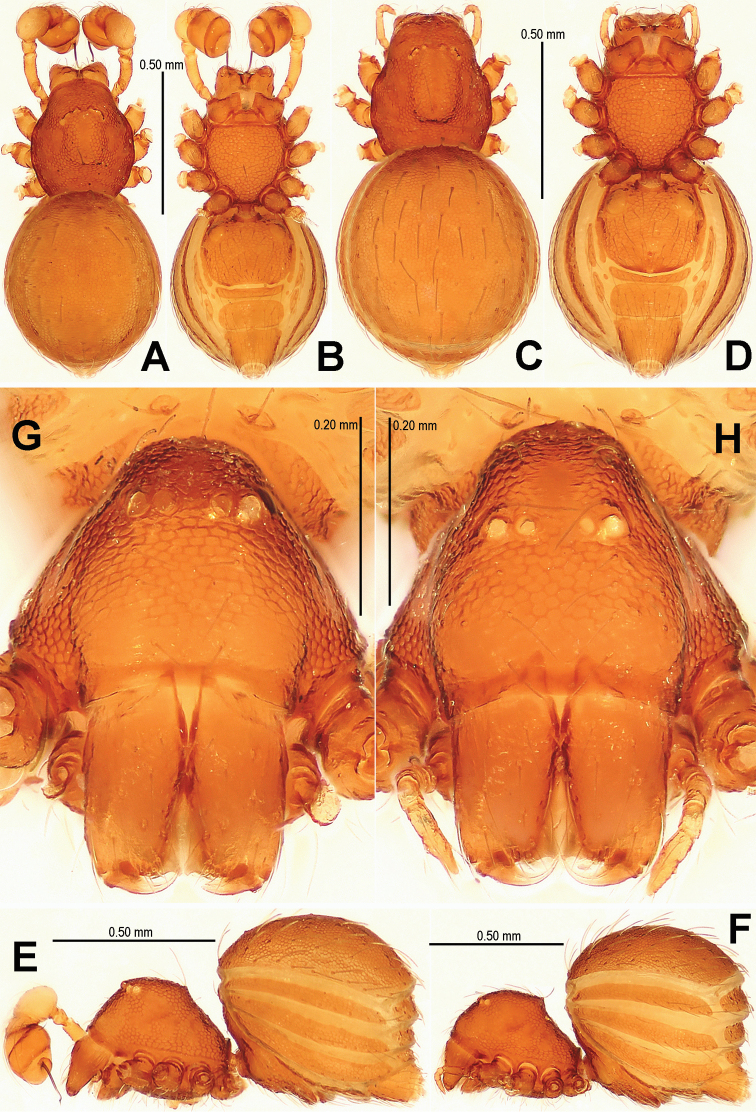
*Singaporemma banxiaoensis* sp. n., male holotype (**A–B, E, G**) and female paratype (**C–D, F, H**). **A–F** Habitus **G, H** Prosoma. **A, C** dorsal view **B, D** ventral view **E, F** lateral view **G, H** anterior view.

**Figure 5.**
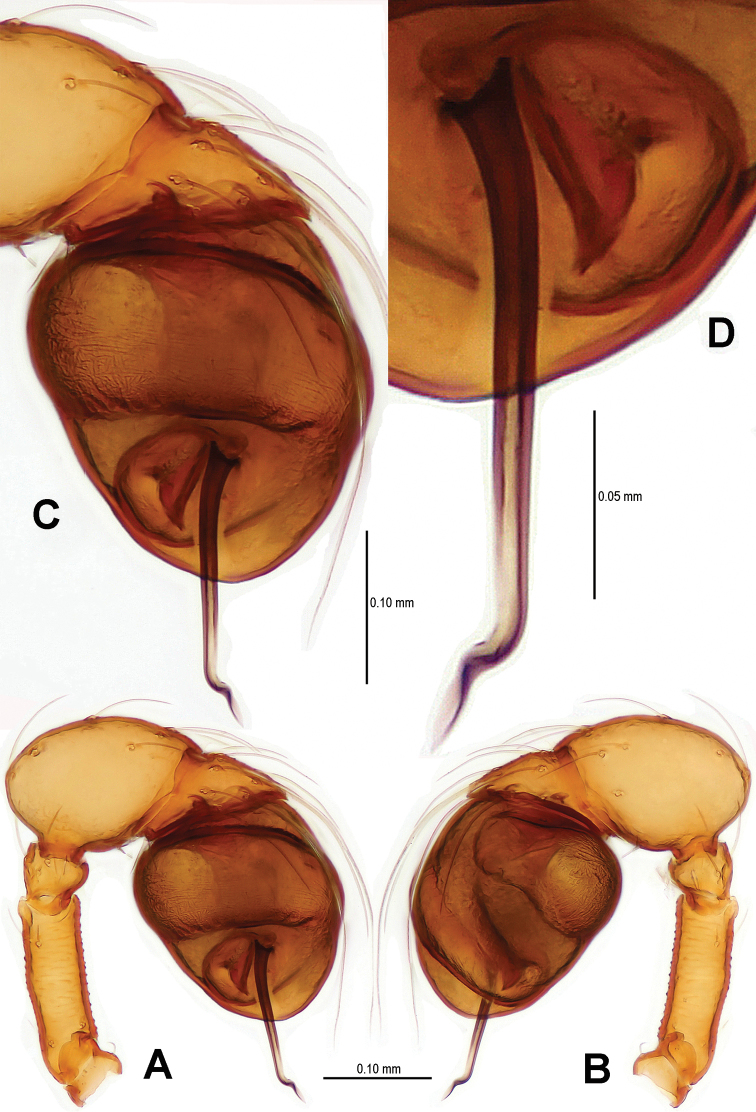
*Singaporemma banxiaoensis* sp. n., male holotype. **A, B** Left palp **C** Bulb **D** Embolus. **A, C–D** prolateral view **B** retrolateral view.

**Figure 6.**
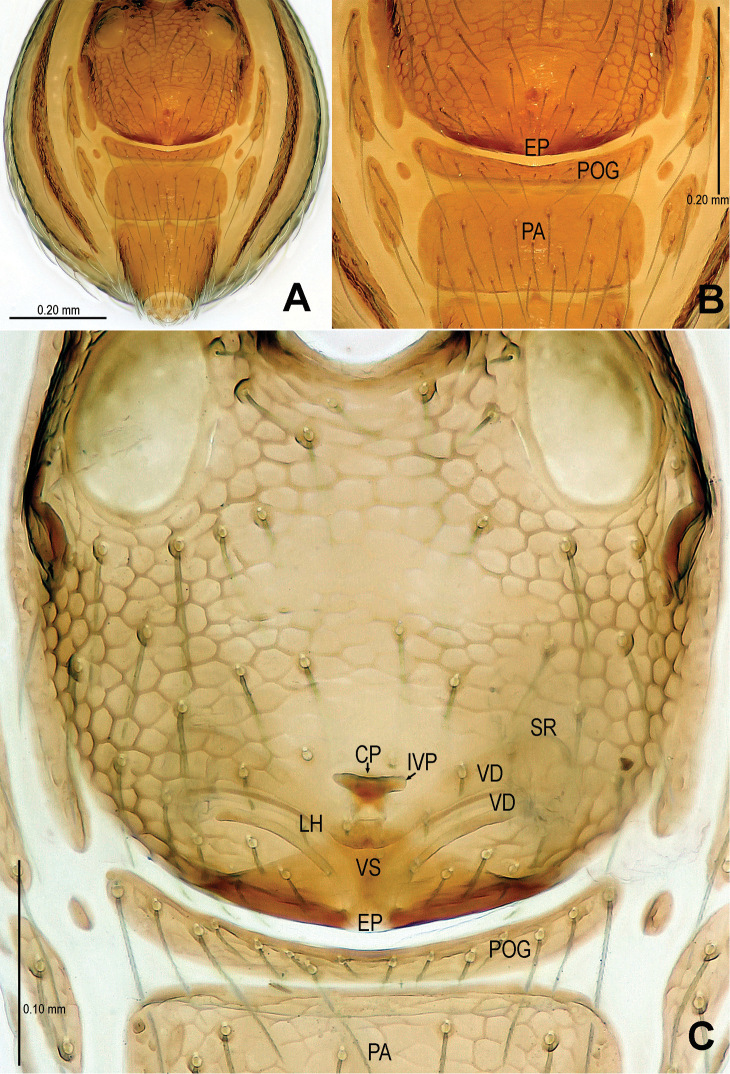
*Singaporemma banxiaoensis* sp. n., female paratype. **A** Opisthosoma **B** Genital area (untreated) **C** Cleared vulva (KOH-treated). **A, B** ventral view **C** dorsal view. Abbrs.: **CP** central process; **EP** epigynal pit; **IVP** inner vulval plate; **LH** lateral horn; **PA** preanal plate; **POG** postgenital plate; **SR** seminal receptaculum; **VD** vulval duct; **VS** vulval stem.

Figs 4 – 6 , 16C–D , 20A , 22

#### Material.

Holotype ♂, paratypes 5♂ and 10♀ (IZCAS), CHINA, Guangxi: Pingxiang City, Xiashi Town, Xinming Village, Banxiaotun, Banxiao Cave, 22°5.542'N, 106°52.148'E, elevation ca. 175 m, 26 July 2011, Xiaoxiao Wang leg.

#### Etymology.

The specific name refers to the type locality; adjective.

#### Diagnosis.

This new species is similar to *Singaporemma halongense* Lehtinen, 1981 (see Lehtinen 1981: 31, figs. 43, 49, 54, 58, 62), but can be distinguished by the white vestigial eyespots in both sexes (Figs 4A, C, G–H), the shape of bulb and psembolus in the male (Figs 5A–D, 16C–D), the inverted triangular inner vulval plate and the wide, robust central process (Figs 6C, 20A), and the presence of a rhombic vulval dorsal plate (Figs 6C, 20A) in the female.

#### Description.

**Male** (holotype). Coloration: body brownish-yellow; legs yellowish-orange.

Measurements: total length 1.02; carapace 0.45 long, 0.36 wide, 0.21 high; abdomen 0.64 long, 0.55 wide, 0.55 high; clypeus 0.16 high; sternum 0.28 long, 0.29 wide. Length of legs: I 1.15 (0.38, 0.12, 0.27, 0.20, 0.20); II 1.13 (0.36, 0.11, 0.27, 0.20, 0.20); III 1.04 (0.30, 0.11, 0.23, 0.20, 0.20); IV 1.35 (0.43, 0.12, 0.34, 0.23, 0.23).

Carapace (Figs 4A–B, G, E) reticulated, margin rugose; eyes white, vestigial; clypeus sloping forward, marginally rounded; cephalic part flat; sternum finely reticulated, margin rugose, with sparse setae. Legs: cuticle striated; all tibiae with 2 trichobothria, and one on metatarsi I–IV.

Abdomen (Figs 4A–B, E): dorsal scutum oval, finely reticulated, covered with sparse setae; ventral scutum reticulated; perigenital scutum present; postepigastral scutum slightly recurved; preanal scutum rectangular.

Palp (Figs 5A–D; 16C, D): femoral cuticle sculptured and granulated, approximate 3 times patella in length; tibia strongly swollen, approx. 2.2 times femur in width (Figs 5A, B); cymbium posteriorly narrow and anteriorly wide (Fig. 5C); bulb egg-shaped, its surface with irregular lines (Fig. 5C); embolus long, proximally straight but distally flexible (Figs 16C–D).

**Female** (paratype). Coloration: same as in male.

Measurements: total length 1.11; carapace 0.47 long, 0.38 wide, 0.22 high; abdomen 0.68 long, 0.64 wide, 0.64 high; clypeus 0.14 high; sternum 0.29 long, 0.29 wide. Lengths of legs: I 1.22 (0.41, 0.13, 0.28, 0.20, 0.21); II 1.15 (0.38, 0.12, 0.27, 0.20, 0.20); III 1.06 (0.32, 0.12, 0.24, 0.20, 0.19); IV 1.41 (0.45, 0.13, 0.36, 0.25, 0.23).

Carapace (Figs 4C–D, H and F) as in male; clypeus slightly lower than in male. Legs: the chaetotaxy as in male.

Abdomen (Figs 4C–D, F; 6A): dorsal scutum reticulated, covered with sparse setae; lateral scutum I anteriorly short, not extending beyond posterior rim of operculum; operculum centrally smooth, laterally rugose; postepigastral scutum slightly curved, medially narrow and laterally wide; perigenital plates long; preanal scutum reticulated, rectangular, with sparse serrated setae.

Genitalia (Figs 6C; 20A): epigynal pit distinct, oval; vulval posterior margin strongly sclerotized (Fig. 6B); vulval dorsal plate rhombic, fused to vulval posterior margin (Figs 6C; 20A); vulval stem triangular (Fig. 20A); vulval ducts proximally narrow, distally wide; lateral horns and vulval ducts forming a “V”-shape; spermathecae rugose, translucent, and membranous; inner vulval plate inverted triangle, distinctly sclerotized, wider than central process (Figs 6C; 20A); central process contracted basally, medially wide (Fig. 20A).

#### Variation.

Total length from 1.00 to 1.07 in males (n = 6) and from 1.05 to 1.14 in females (n = 10).

#### Distribution.

Known only from the type locality (Fig. 22).

### Singaporemma wulongensis sp. n.

http://zoobank.org/6FF00E16-1A9B-4497-BFC7-317B0B9A403A

http://species-id.net/wiki/Singaporemma_wulongensis

**Figure 7.**
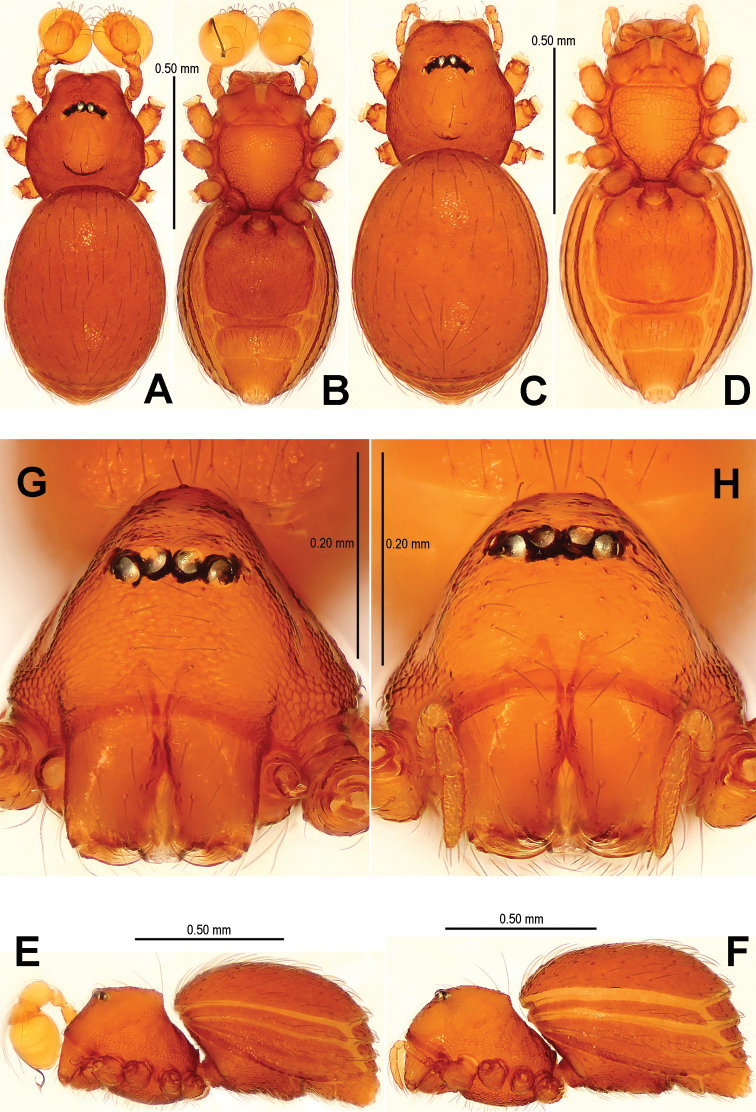
*Singaporemma wulongensis* sp. n., male holotype (**A–B, E, G**) and female paratype (**C–D, F, H**). **A–F** Habitus **G–H** Prosoma. **A, C** dorsal view **B, D** ventral view **E, F** lateral view **G, H** anterior view.

**Figure 8.**
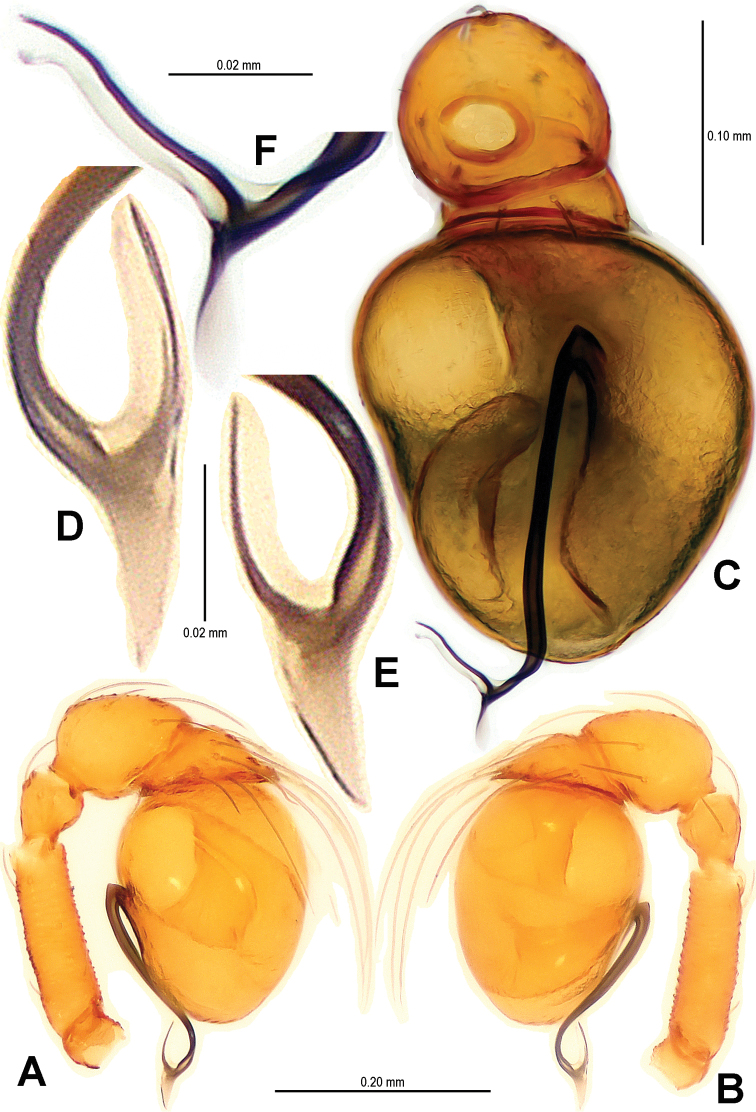
*Singaporemma wulongensis* sp. n., male holotype. **A, B** Left palp **C** Bulb (KOH-treated) **D–F** Distal embolus. **A, E** prolateral view **B, D** retrolateral view **C, F** posterior view.

**Figure 9.**
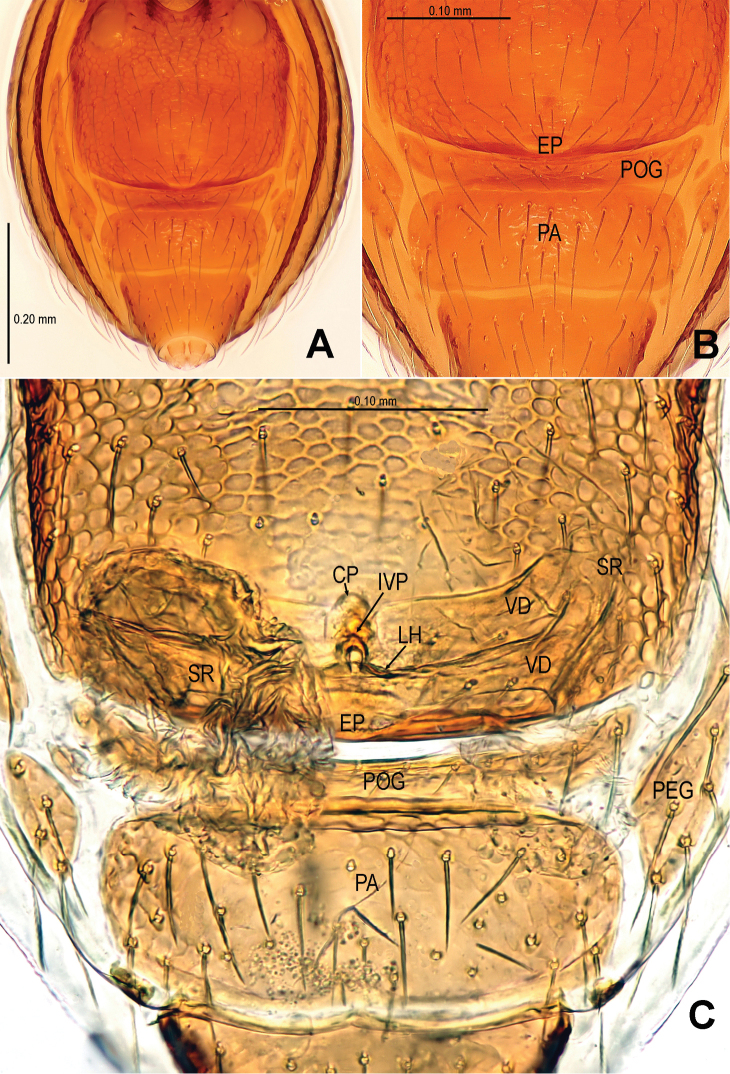
*Singaporemma wulongensis* sp. n., female paratype. **A** Opisthosoma **B** Genital area (untreated) **C** Cleared vulva (KOH-treated). **A, B** ventral view **C** dorsal view. Abbrs.: **CP** central process; **EP** epigynal pit; **IVP** inner vulval plate; **LH** lateral horn; **PA** preanal plate; **POG** postgenital plate; **SR** seminal receptaculum; **VD** vulval duct; **VS** vulval stem.

Figs 7 – 9 , 17 , 20B , 22

#### Material.

Holotype ♂, paratypes 8♂ and 20♀ (SCUM), CHINA, Chongqing: Wulong County, Tudi Town, Tiansheng Village, Xiaodong Cave, 29°31.853'N, 107°50.817'E, elevation ca. 1050 m, 17 October 2010, Liang Dou and Yucheng Lin leg.

#### Etymology.

The specific name refers to the type locality; adjective.

#### Diagnosis.

This new species is similar to *Singaporemma bifurcata* Lin & Li, 2010 (see Lin & Li 2010: figs 29–37) but the male can be distinguished by the base of embolus (Figs 8A–C, 17A–C), the flexible embolic end (Figs 8D–F, 17D–E), and the long oval palpal bulb (Figs 8A–B, 17A–B). The females are distinguished by the “Ω”-shaped inner vulval plate, and the long central process (Figs 9C, 20B).

#### Description.

**Male** (holotype). Coloration: body reddish-brown; legs yellowish-brown.

Measurements: total length 1.21; carapace 0.53 long, 0.45 wide, 0.44 high; abdomen 0.79 long, 0.60 wide, 0.54 high; clypeus 0.18 high; sternum 0.32 long, 0.32 wide. Length of legs: I 1.29 (0.41, 0.13, 0.30, 0.21, 0.23); II 1.16 (0.36, 0.13, 0.27, 0.20, 0.21); III 1.07 (0.32, 0.13, 0.23, 0.19, 0.21); IV 1.43 (0.43, 0.13, 0.36, 0.25, 0.26).

Carapace (Figs 7A–B, G and E) finely reticulated, margin rugose; eyes with black base; cephalic part flat, covered with long setae; clypeus sharply sloping anteriorly; sternum reticulated, marginally rugose. Legs: cuticle striated; all tibiae with 3 trichobothria and all metatarsi with a trichobothrium.

Abdomen (Figs 7A–B, E): dorsal scutum long, oval, covered with long setae, margin reticulated, center granulated; ventral scutum reticulated, margin rugose; lateral scutum I short, perigenital plate broad.

Palp (Figs 8A–F; 17A–E): femoral cuticle granular, striated, approx. 3 times as long as patella; patella proximally narrow, distally wide; tibia short, swollen, 1.6 times as wide as femur; bulb egg-shaped, surface smooth; embolus long, strongly sclerotized, starting from subproximal-ventral 1/3 position of bulbous surface, and curved downwards (Figs 8C; 17A, C); embolic tip flexuous, forked (Figs 8D–F; 17D–E).

**Female** (paratype). Coloration: body slightly lighter than in male; legs yellowish-brown.

Measurement: total length 1.23; carapace 0.54 long, 0.43 wide, 0.41 high; abdomen 0.80 long, 0.63 wide, 0.59 high; clypeus 0.17 high; sternum 0.30 long, 0.31 wide. Length of legs: I 1.21 (0.38, 0.13, 0.29, 0.20, 0.21); II 1.11 (0.34, 0.13, 0.25, 0.18, 0.21); III 1.03 (0.30, 0.12, 0.22, 0.18, 0.21); IV 1.38 (0.42, 0.13, 0.34, 0.23, 0.25).

Carapace (Figs 7C–D, F and H) as in male, except for clypeal area smooth. Legs: chaetotaxy as in male.

Abdomen (Figs 7C–D, F; 9A): as in male, except for lighter coloration; lateral scutum I anteriorly short, not extending beyond the posterior rim of operculum; perigenital plate broad; postepigastral scutum curved, its posterior margin overlapped with anterior margin of preanal scutum; preanal scutum regularly rectangular, width equal to 2 times length, surface covered with serrated setae.

Genitalia (Figs 9B–C; 20B): genital area smooth; epigynal folds absent, genital basal margin sclerotized (Fig. 9B); epigynal pit small, oval, transverse; vulval stem absent; lateral horns contracted, supporting the base of vulval ducts; inner vulval plate “Ω”-shaped, slightly sclerotized, shorter and narrower than central process (Figs 9C; 20B); central process large, translucent; vulval duct wide; spermathecae large, rugose. (Fig. 20B).

#### Variation.

Total length from 1.14 to 1.25 in males (n = 9) and from 1.16 to 1.30 in females (n = 20).

#### Distribution.

Known only from the type locality (Fig. 22).

### Tetrablemma

O. P.-Cambridge, 1873

http://species-id.net/wiki/Tetrablemma

#### Type species.

*Tetrablemma medioculatum* O. P.-Cambridge, 1873 from Sri Lanka.

#### Diagnosis.

*Tetrablemma* is close to *Singalangia* Lehtinen, 1981 and *Rhinoblemma* Lehtinen, 1981. *Tetrablemma* is separated from *Singalangia* by largely different pattern of abdominal plates, by well developed lateral horns of vulva, and by lack of apomorphic modifications of the same type in sternum and epigynal area. It is separated from *Rhinoblemma* by different types of sexual dimorphism in the male clypeal area and chelicerae.

#### Distribution.

Twenty five species have previously been described from Angola, Western Australia, China, Flores, India, Laos, Micronesia, Myanmar, Nepal, St. Helena, Queensland, Samoa, Seychelles, Sri Lanka, Sulawesi, Sumatra, Trinidad, Victoria, and Vietnam, collected mainly from forest litter or caves.

### Tetrablemma menglaensis sp. n.

http://zoobank.org/22DDEE3E-1084-45A3-9BE2-484281B16A9B

http://species-id.net/wiki/Tetrablemma_menglaensis

**Figure 10.**
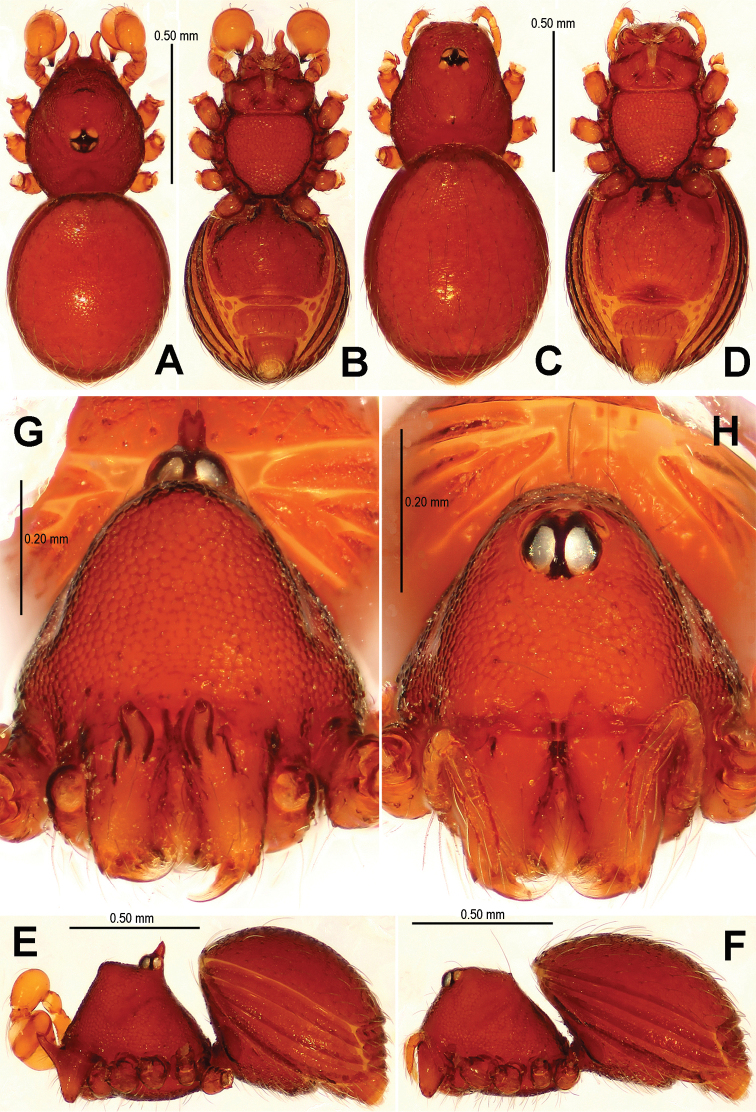
*Tetrablemma menglaensis* sp. n., male holotype (**A–B, E, G**) and female paratype (**C–D, F, H**). **A–F** Habitus **G, H** Prosoma. **A, C** dorsal view **B, D** ventral view **E, F** lateral view **G, H** anterior view.

**Figure 11.**
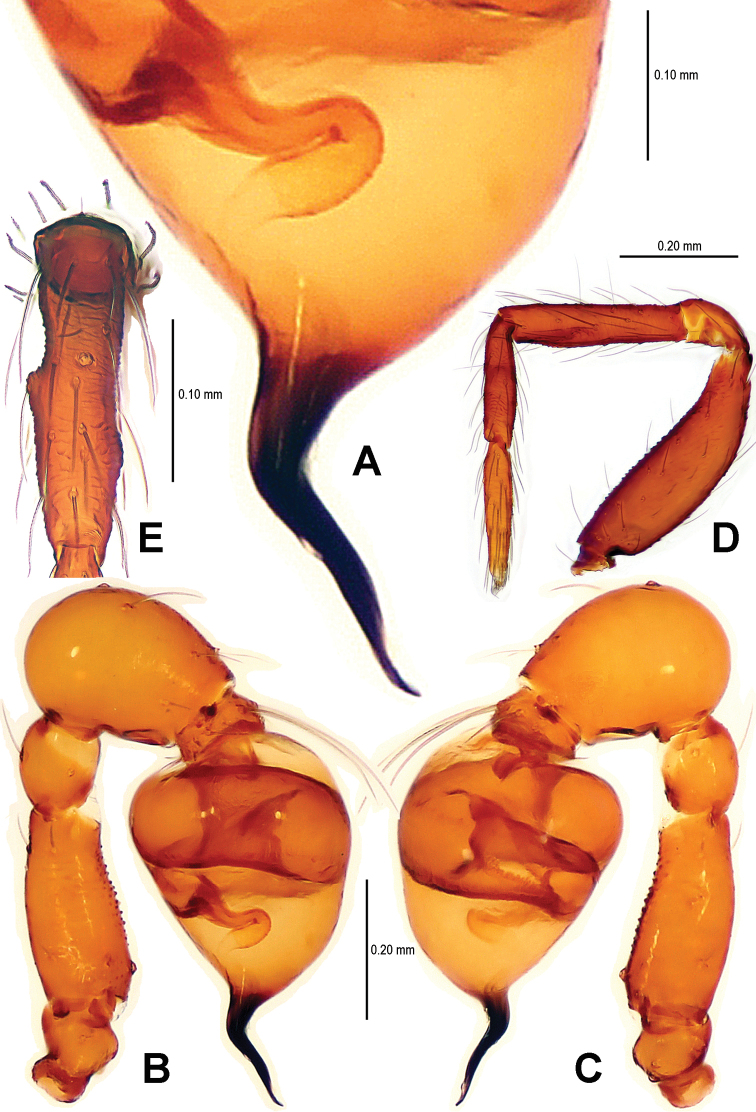
*Tetrablemma menglaensis* sp. n., male holotype. **A** Embolus and sperm duct **B, C** Left palp **D** Left leg I **E** Left metatarsus I. **A, B** prolateral view **C, D** retrolateral view **E** anterior view.

**Figure 12.**
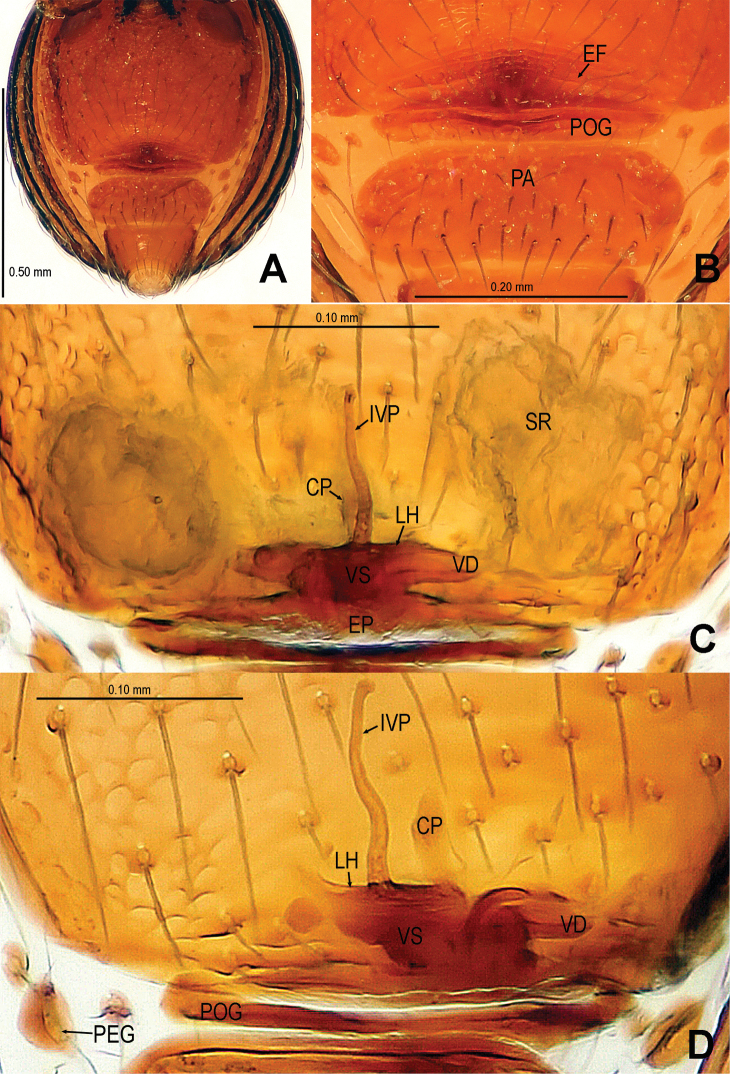
*Tetrablemma menglaensis* sp. n., female paratype. **A** Opisthosoma **B** Genital area (untreated) **C, D** Cleared vulva (KOH-treated). **A, B** ventral view **C** dorsal view **D** dorsal-lateral view. Abbrs.: **CP** central process; **EF** epigynal fold; **EP** epigynal pit; **IVP** inner vulval plate; **LH** lateral horn; **PA** preanal plate; **POG** postgenital plate; **SR** seminal receptaculum; **VD** vulval duct; **VS** vulval stem.

Figs 10 – 12 , 18C–D , 21 , 22

#### Material.

Holotype ♂, paratypes 2♀ (SCUM), CHINA, Yunnan: Mengla County, Mengyuan Town, Chengzi Village, Yeniudong Scenery Spot, Yeniu 2^#^ Cave, 21°43.208'N, 101°23.294'E, elevation ca. 760 m, 16 August 2011, Yucheng Lin and Guo Zheng leg.

#### Etymology.

The specific name refers to the type locality; adjective.

#### Diagnosis.

The male of this new species is similar to *Tetrablemma namkhan* Lin & Li, 2012 (see Lin and Li 2012: figs 4A–G, 5A–C, 6A–C), *Tetrablemma loebli* Bourne, 1980 (see Lehtinen 1981: figs 219, 221, 223–224), *Tetrablemma marawula* Lehtinen, 1981 (see Lehtinen 1981: figs 255, 266), and *Tetrablemma brevidens* Tong & Li, 2008 (see Tong and Li 2008: figs 5A, C, F–I), but can be distinguished by a forked cephalic tubercle (Figs 10E, G), a crooked cheliceral horn (Figs 10A–B, E), the swollen palpal tibia (Figs 11B–C, 18C), the course of sperm duct, and the long-tongue shaped embolus (Figs 11A, 18D). The female is similar to *Tetrablemma nandan* Lin & Li, 2010 (see Lin and Li 2010: figs 46–49) and *Tetrablemma marawula* Lehtinen, 1981 (see Lehtinen 1981: figs 256, 283), but can be recognized by the narrow postepigastral scutum (Figs 12A–B), the long S-shaped inner vulval plate (Figs 12C–D, 21A–B), the absence of vulval dorsal plate, and the wide central process (Figs 12D, 21B).

#### Description.

**Male** (holotype). Coloration: body reddish-brown; legs yellowish-brown.

Measurements: total length 1.18; carapace 0.54 long, 0.46 wide, 0.36 high; abdomen 0.98 long, 0.63 wide, 0.52 high; clypeus 0.27 high. Sternum 0.31 long, 0.34 wide. Length of legs: I 1.27 (0.38, 0.14, 0.30, 0.21, 0.23); II 1.21 (0.38, 0.13, 0.29, 0.21, 0.22); III 1.11 (0.34, 0.11, 0.25, 0.20, 0.21); IV 1.40 (0.43, 0.11, 0.36, 0.26, 0.25).

Carapace (Figs 10A, E and G) completely reticulate, margin rugose; ocular area with a short, bifurcate tubercle; clypeal area distinctly convex, margin rounded; cheliceral horn narrow, medially curved in dorsal view; sternum centrally reticulated, marginally sclerotized and rugose. Legs: cuticle sculptured; femur I slightly swollen; all tibiae with 2 trichobothria, and one on metatarsi I–IV; metatarsus I with two small lateral tubercles (Figs 11D, E).

Abdomen (Figs 10A–B, E): dorsal scutum short, oval, finely granulated; ventral scuta reticulated and striated; lateral scutum I short; postepigastral scutum short, narrower than preanal scutum (Fig. 10B).

Palp (Figs 11A–C; 18C–D): femur slightly swollen, ventrally granulated; patella short, approx. as 1/2 long as femur; tibia smooth, swollen, approx. 2 times as wide as patella; bulb pyriform, smooth; embolus long, bent, strongly sclerotized; sperm duct visible through bulbal integument (Figs 11A–C; 18C, D).

**Female** (paratype). Coloration and modifications as in male, but cephalic tubercle and cheliceral horn absent.

Measurements: total length 1.27; carapace 0.58 long, 0.45 wide, 0.28 high; clypeus 0.19 high; sternum 0.32 long, 0.34 wide; abdomen 0.91 long, 0.68 wide, 0.50 high. Length of legs: I 1.29 (0.41, 0.13, 0.31, 0.21, 0.23); II 1.21 (0.38, 0.13, 0.29, 0.21, 0.22); III 1.12 (0.34, 0.12, 0.25, 0.20, 0.21); IV 1.47 (0.46, 0.13, 0.38, 0.27, 0.24).

Carapace (Figs 10C, F and H): reticulation as in male; clypeal area nearly vertical anteriorly; cephalic part flat; cheliceral frontal surface with a small basal tubercle; sternum as in male. Legs: chaetotaxy and number of trichobothria as in male.

Abdomen (Figs 10C–D, F; 12A): lateral scutum I anteriorly short, not extending beyond the posterior rim of operculum; postgenital plate straight, narrower than preanal scutum; preanal scutum wider than long, anterior margin rugose (Fig. 21A), covered with serrated setae (Fig. 12B).

Genitalia (Figs 12B–D; 21A–B): epigynal folds laterally narrow, medially wide (Fig. 12B); epigynal pit narrow, indistinct, separated with vulval stem and lateral horns; vulval stem strongly sclerotized (Figs 12C; 21A); spermathecae rugose, membranous; lateral horns wide, strong, supporting the base of vulval ducts of seminal receptacle; inner vulval plate S-shaped, very long, at least 2 times longer than central process (Figs 12D; 21B); central process wide, basally contracted (Fig. 21B); vulval duct narrow, connected with lateral horn and spermathecae.

#### Distribution.

Known only from the type locality (Fig. 22).

### Tetrablemma ziyaoensis sp. n.

http://zoobank.org/38AA57F1-7F11-46A4-A2AA-6A259B2A52D5

http://species-id.net/wiki/Tetrablemma_ziyaoensis

**Figure 13.**
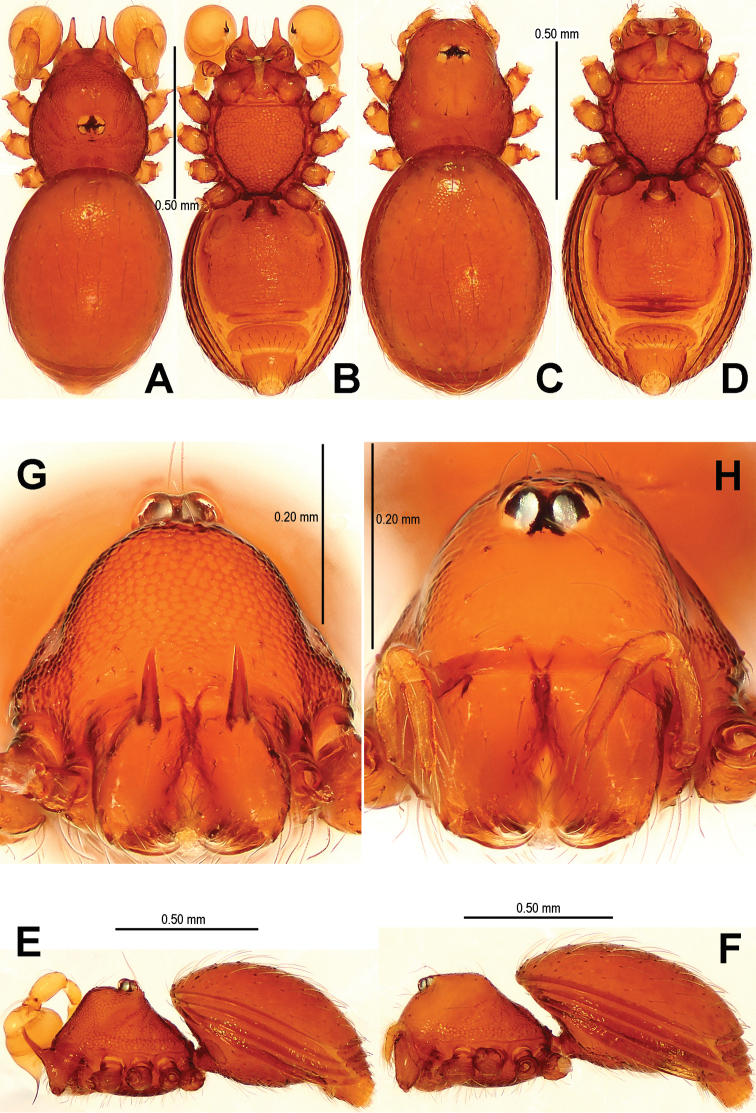
*Tetrablemma ziyaoensis* sp. n., male holotype (**A–B, E, G**) and female paratype (**C–D, F, H**). **A–F** Habitus **G, H** Prosoma. **A, C** dorsal view **B, D** ventral view **E, F** lateral view **G, H** anterior view.

**Figure 14.**
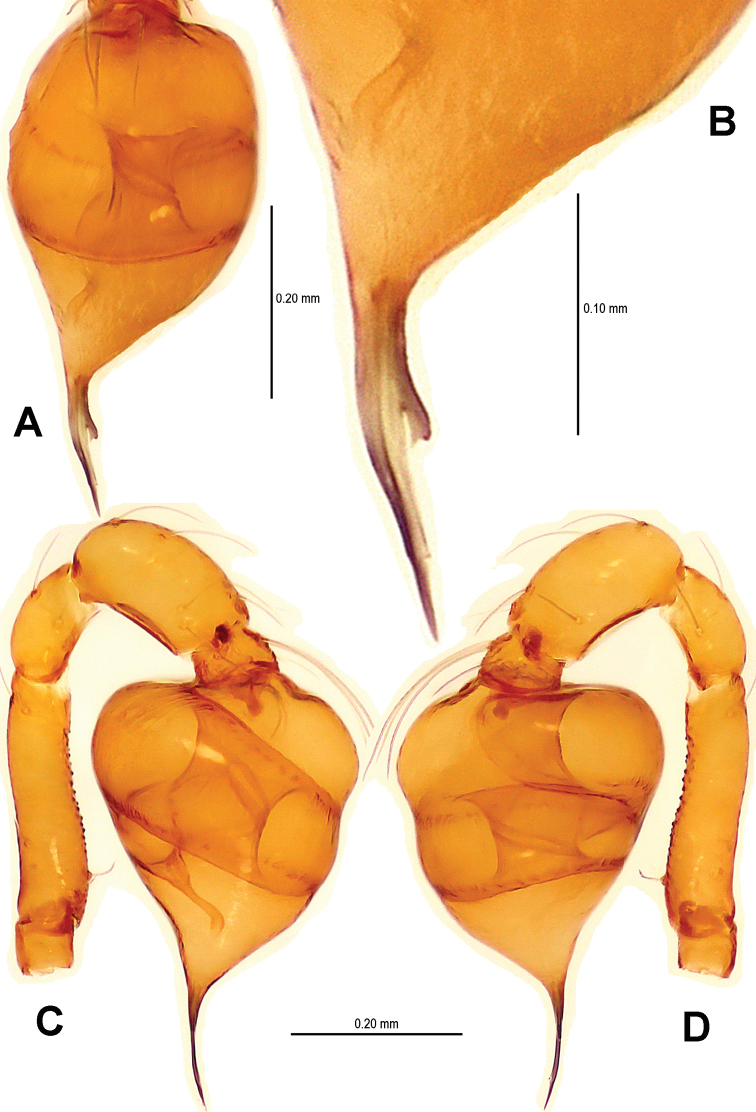
*Tetrablemma ziyaoensis* sp. n., male holotype. **A** Bulb **B** Embolus and spermic duct**C, D** Left palp. **A, B** anterior view **C** prolateral view **D** retrolateral view.

**Figure 15.**
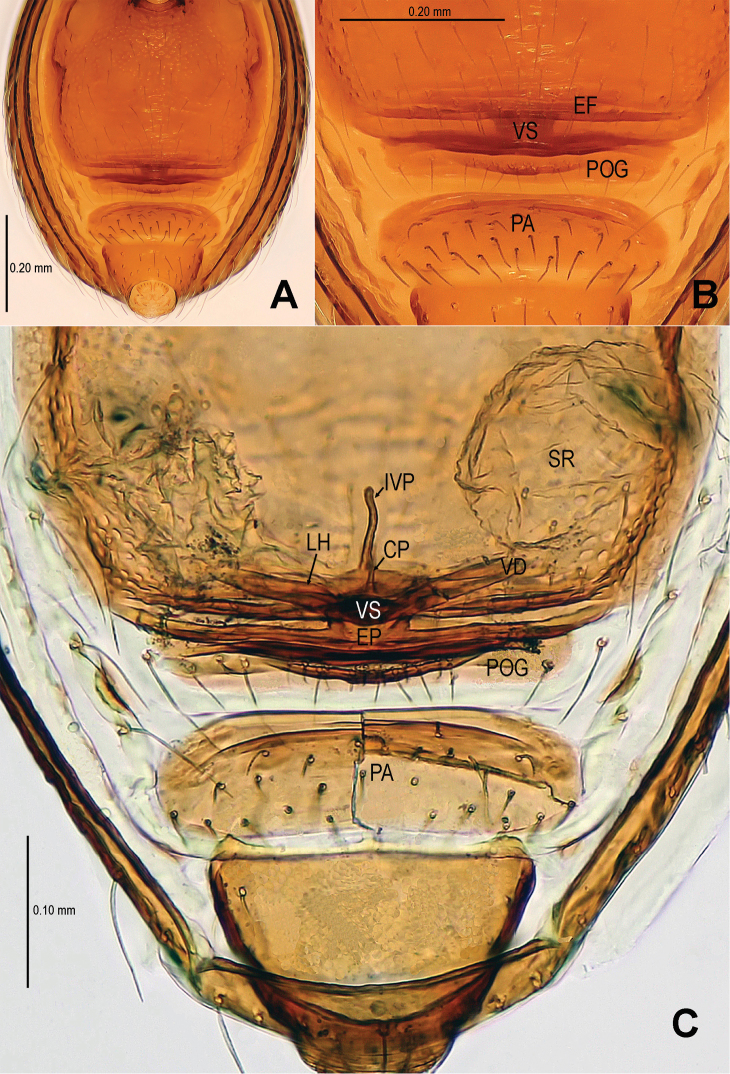
*Tetrablemma ziyaoensis* sp. n., female paratype. **A** Opisthosoma **B** Genital area (untreated) **C** Cleared vulva (KOH-treated). **A, B** ventral view **C** dorsal view. Abbrs.: **CP** central process; **EF** epigynal fold; **EP** epigynal pit; **IVP** inner vulval plate; **LH** lateral horn; **PA** preanal plate; **PEG** perigenital plate; **POG** postgenital plate; **SR** seminal receptaculum; **VD** vulval duct; **VS** vulval stem.

Figs 13 – 15 , 18A–B , 19A , 22

#### Material.

Holotype ♂, paratypes 1♂ and 1♀ (IZCAS), CHINA, Guangxi: Chongzuo City, Fusui County, Dongmen Town, Ziyao Village, Yinhe Cave, 22°19.763'N, 107°47.526'E, elevation ca. 154 m, 13 July 2011, Xiaoxiao Wang leg.

#### Etymology.

The specific name refers to the type locality; adjective.

#### Diagnosis.

This new species is similar to *Tetrablemma thamin* Labarque & Grismado, 2009 (see Labarque and Grismado 2009: figs 1–4, 6–11), *Tetrablemma marawula* (see Lehtinen 1981: 61, figs 255–256, 266, 281), and *Tetrablemma manggarai* Lehtinen, 1981 (see Lehtinen 1981: 61, figs 259–260, 269, 282a, 287), but males can be distinguished by the tapering cheliceral horn, the no swollen palpal tibia, the deflective pear-shape bulb (Figs 14C, D) and the medial forked embolus (Figs 14A, B, 18B vs. Labarque and Grismado 2009: figs 1, 3, but not forked in *Tetrablemma marawula* and *Tetrablemma manggarai*). Females differ by the presence of a triangular dorsal plate (absent in *Tetrablemma thamin*, *Tetrablemma marawula* and *Tetrablemma manggarai*), the basally contracted central plate, the wide postepigastral scutum, and the presence of a long and distinct anterior fold on the preanal scutum (Figs 15B, C; 19A) (absent in *Tetrablemma thamin* and *Tetrablemma marawula*, unknown in *Tetrablemma manggarai*).

**Description. Male** (holotype). Coloration: body yellowish-brown; legs light brown.

Measurements: total length 1.13; carapace 0.50 long, 0.39 wide, 0.26 high; abdomen 0.75 long, 0.55 wide, 0.44 high; clypeus 0.26 high; sternum 0.29 long, 0.30 wide. Length of legs: I 1.29 (0.41, 0.13, 0.30, 0.22, 0.23); II 1.18 (0.38, 0.11, 0.27, 0.21, 0.22); III 1.07 (0.32, 0.11, 0.23, 0.20, 0.21); IV 1.47 (0.45, 0.13, 0.38, 0.29, 0.24).

Carapace (Figs 13A–B, E and G): most of surface reticulated, marginally rugose; ocular area slightly raised, located at center, with two long setae between PLEs; clypeal area distinctly convex, margin rounded; cheliceral horn thin, sharp, distally curved; sternum finely reticulated, strongly sclerotized at margin. Legs: cuticle striated and granular; femur I not swollen; tibiae I–III with 3 trichobothria, 4 on tibia IV, and metatarsi I–IV with one trichobothrium.

Abdomen (Figs 13A–B, E): dorsal scutum short, oval, slightly granulated; ventral episgastric scutum centrally reticulated, laterally striated; lateral scutum I short; postepigastral scutum narrow, approximate 1/2 times width of preanal scutum, but same length.

Palp (Figs 14A–D; 18A–B): femur ventrally granulated (Figs 14C, D); tibia smooth, not swollen, approx. as long as 2/3 times femur; bulb deflective pear-shape, smooth; embolus long, needle-like, slightly sclerotized, medially forked (Figs 14A, B; 18B).

**Female** (paratype). Coloration as in male.

Measurements: total length 1.21; carapace 0.54 long, 0.41 wide, 0.23 high; abdomen 0.84 long, 0.63 wide, 0.45 high; clypeus 0.16 high; sternum 0.30 long, 0.31 wide. Length of legs: I 1.36 (0.45, 0.13, 0.30, 0.23, 0.25); II 1.25 (0.39, 0.12, 0.28, 0.22, 0.24); III 1.13 (0.32, 0.13, 0.23, 0.21, 0.23); IV 1.54 (0.47, 0.13, 0.39, 0.29, 0.25).

Carapace (Figs 13C–D, F and H): cephalic part flat, smooth; ocular area anterior; clypeal area smooth, anteriorly upright; thoracic area reticulated; chelicerae with a small baso-lateral tubercle; sternum reticulated, covered with sparse setae. Legs: chaetotaxy and number of trichobothria as in male.

Abdomen (Figs 13C–D, F; 15A): lateral scutum I anteriorly long, extending beyond the anterior rim of operculum; ventral episgastric scutum reticulated, laterally rugose; postepigastral scutum straight, medially narrow, laterally wide, as wide as preanal scutum; preanal scutum long oval, covered with serrated setae, a distinct furrow near anterior margin (Fig. 15B).

Genitalia (Figs 15B–C; 19A): epigynal fold wide (Fig. 15B), epigynal pit small, rift-shaped; vulval stem wide “V”-shaped, strongly sclerotized; lateral horns slightly sclerotized, supporting the base of vulval ducts of spermathecae; dorsal plate broad, triangular, distal margin extending to form a long inner vulval plate; inner vulval plate narrow, slightly sclerotized, longer than central process (Figs 15C; 19A); central process contracted at base (Fig. 19A).

#### Distribution.

Known only from the type locality (Fig. 22).

**Figure 16.**
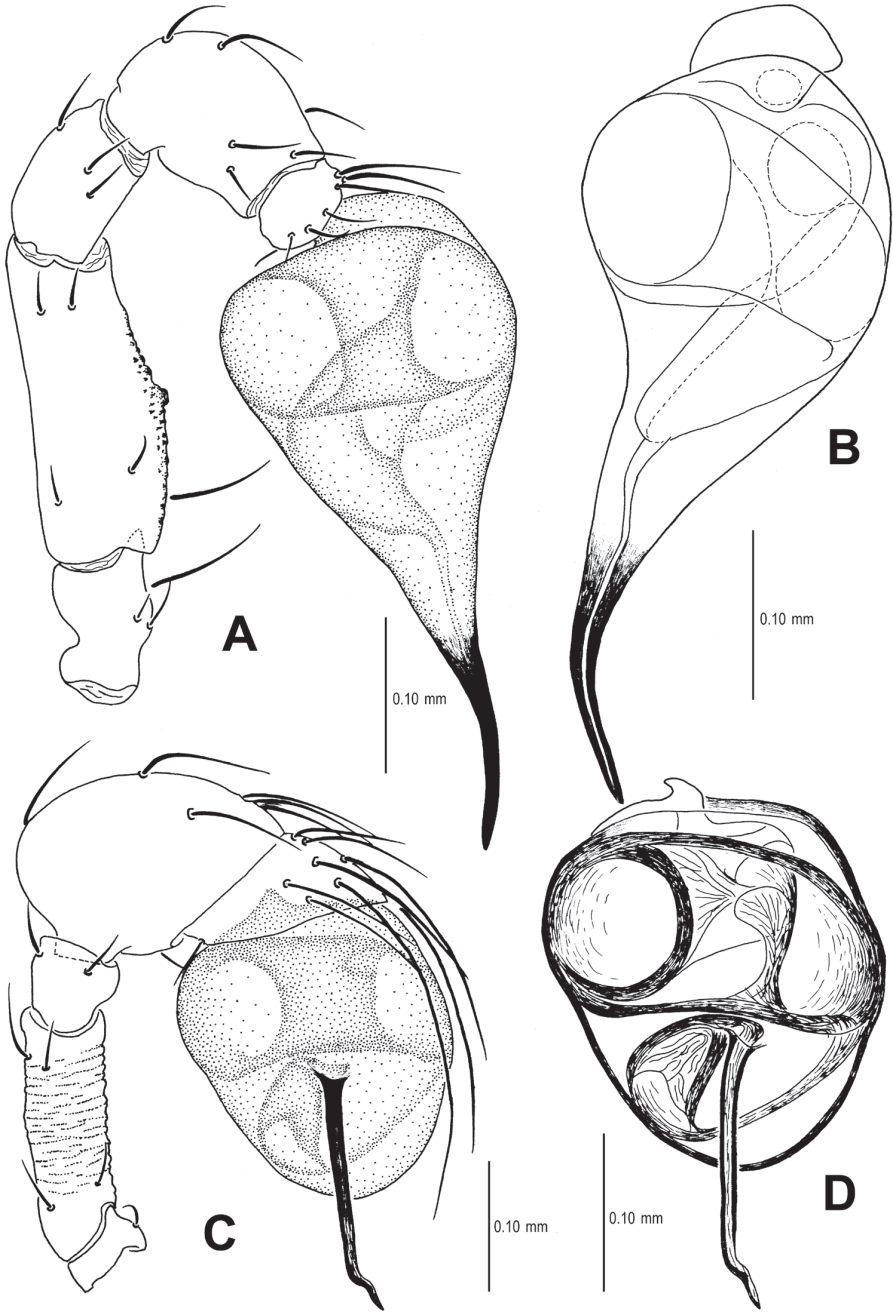
*Sinamma oxycera* gen. n. & sp. n., male holotype (**A, B**) *Singaporemma banxiaoensis* sp. n., male holotype (**C, D**). **A, C** Left palp **B, D** Bulb and spermic duct (lactic acid-treated). **A, C–D** prolateral view **B** anterior view.

**Figure 17.**
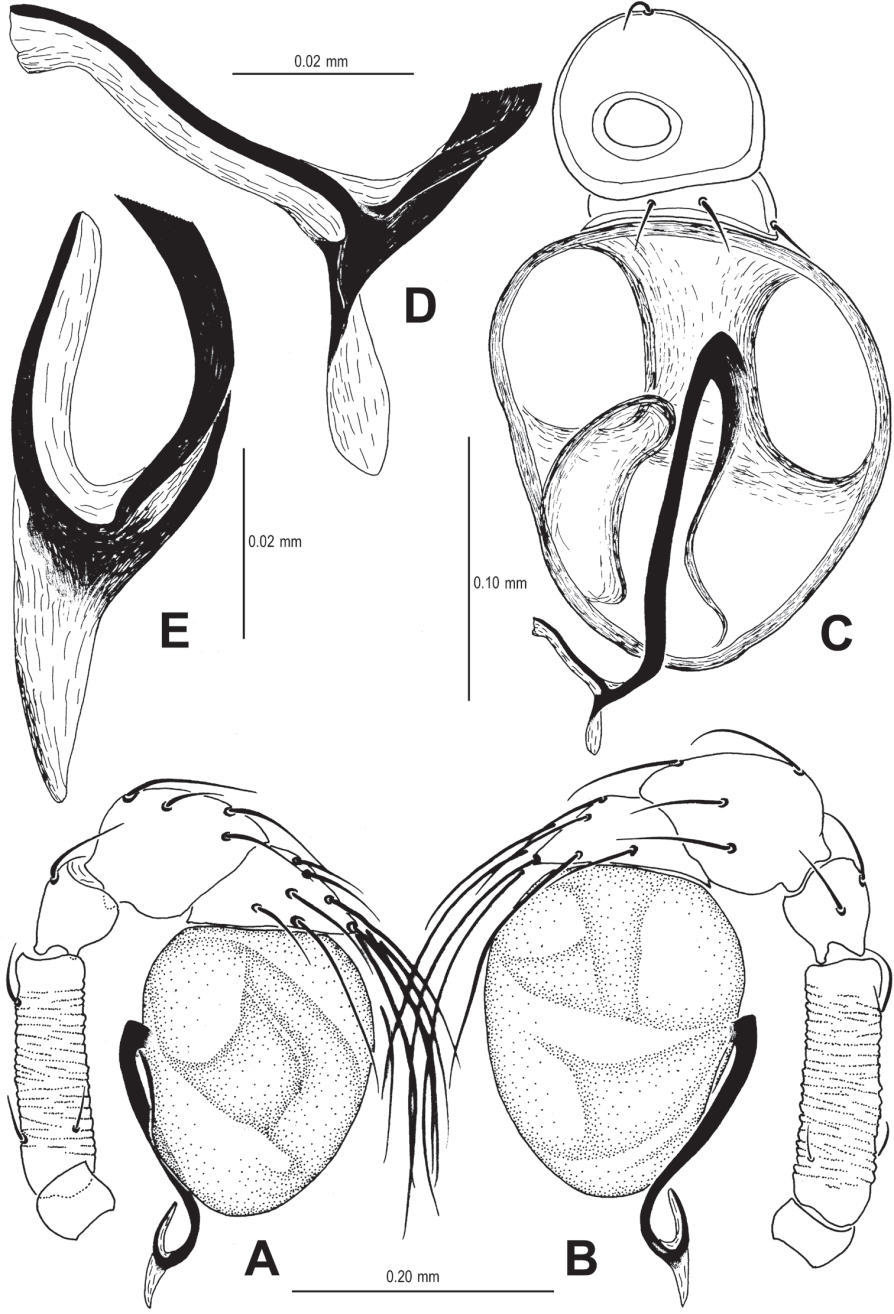
*Singaporemma wulongensis* sp. n., male holotype. **A, B** Left palp **C** Bulb and spermic duct (lactic acid-treated) **D, E** Tip of embolus. **A, E** prolateral view **B** retrolateral view **C, D** posterior view.

**Figure 18.**
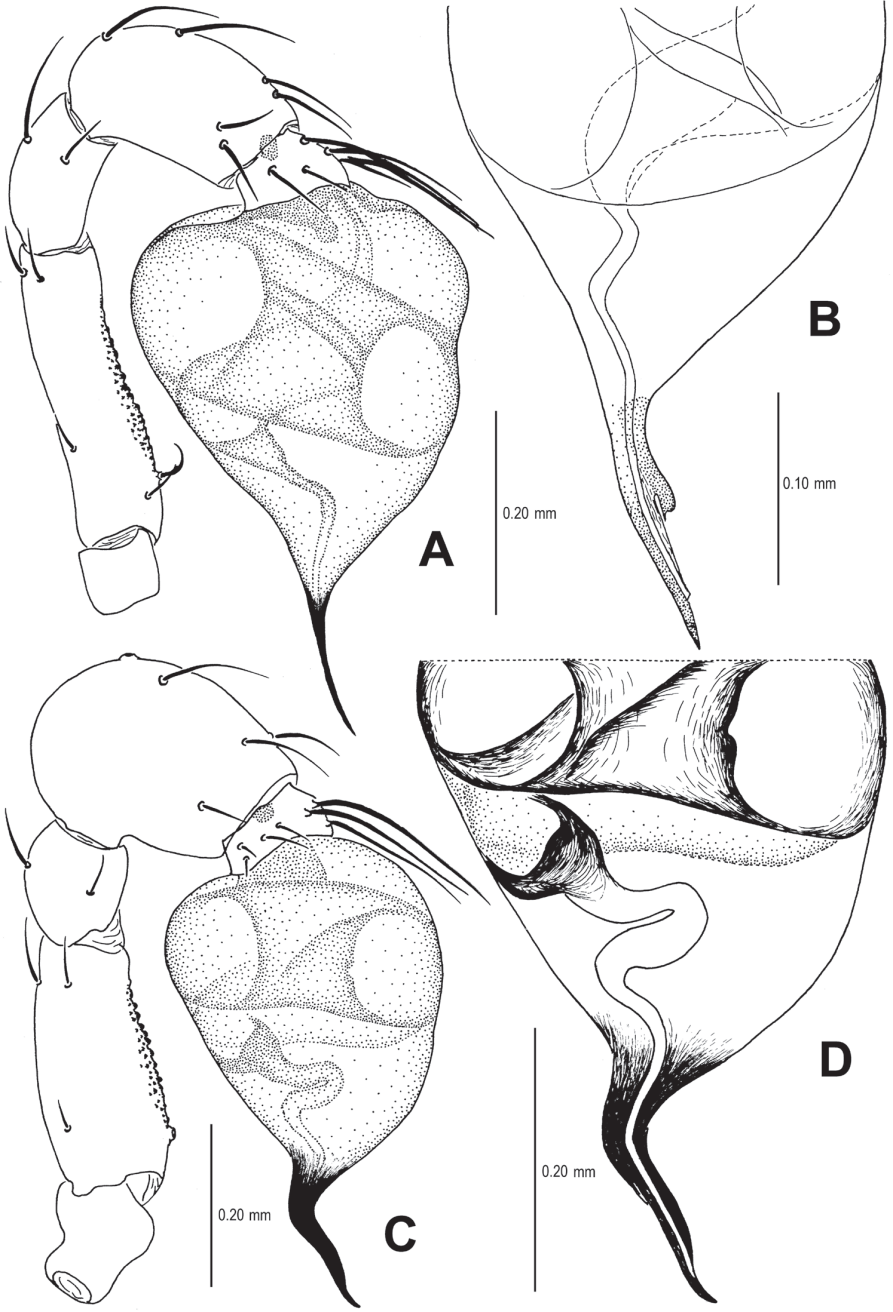
*Tetrablemma ziyaoensis* sp. n., male holotype (**A, B**) *Tetrablemma menglaensis* sp. n., male holotype (**C, D**). **A, C** Left palp **B, D** Bulb and spermic duct (lactic acid-treated). **A, C–D** prolateral view **B** anterior view.

**Figure 19.**
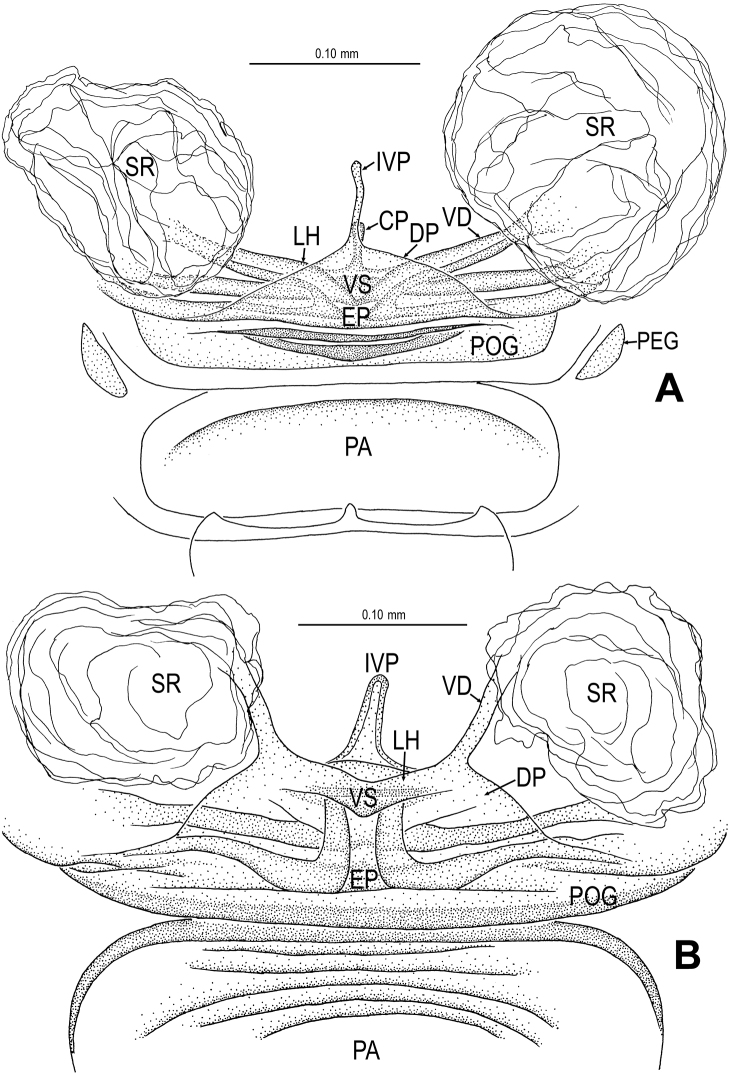
*Tetrablemma ziyaoensis* sp. n., female paratype (**A**) *Sinamma oxycera* gen. n. & sp. n., female paratype (**B**). **A, B** Cleared vulva (KOH-treated), dorsal view. Abbrs.: **CP** central process; **DP** dorsal plate; **EP** epigynal pit; **IVP** inner vulval plate; **LH** lateral horn; **PA** preanal plate; **PEG** perigenital plate; **POG** postgenital plate; **SR** seminal receptaculum; **VD** vulval duct; **VS** vulval stem.

**Figure 20.**
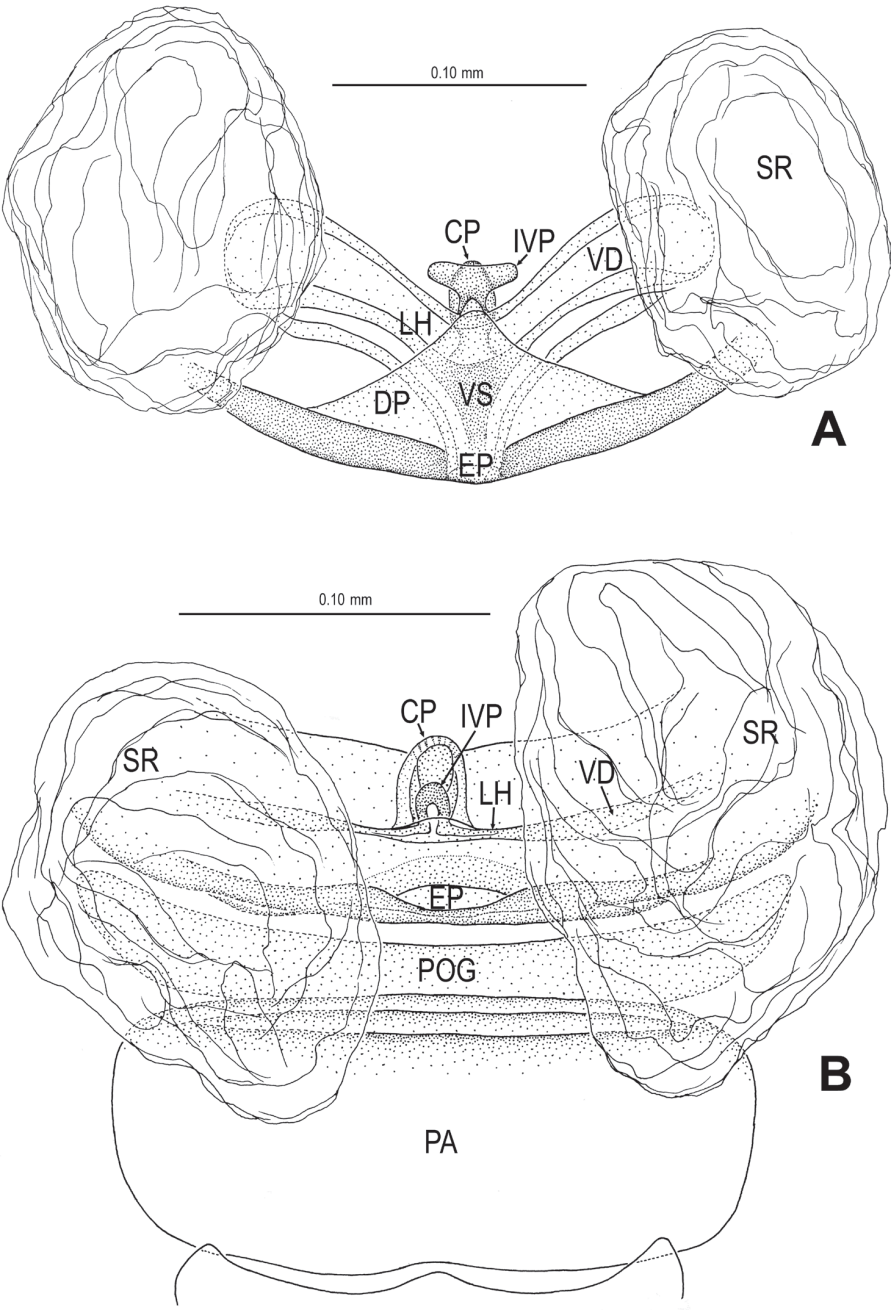
*Singaporemma banxiaoensis* sp. n., female paratype (**A**) *Singaporemma wulongensis* sp. n., female paratype (**B**). **A, B** Cleared vulva (KOH-treated), dorsal view. Abbrs.: **CP** central process; **DP** dorsal plate; **EP** epigynal pit; **IVP** inner vulval plate; **LH** lateral horn; **PA** preanal plate; **POG** postgenital plate; **SR** seminal receptaculum; **VD** vulval duct; **VS** vulval stem.

**Figure 21.**
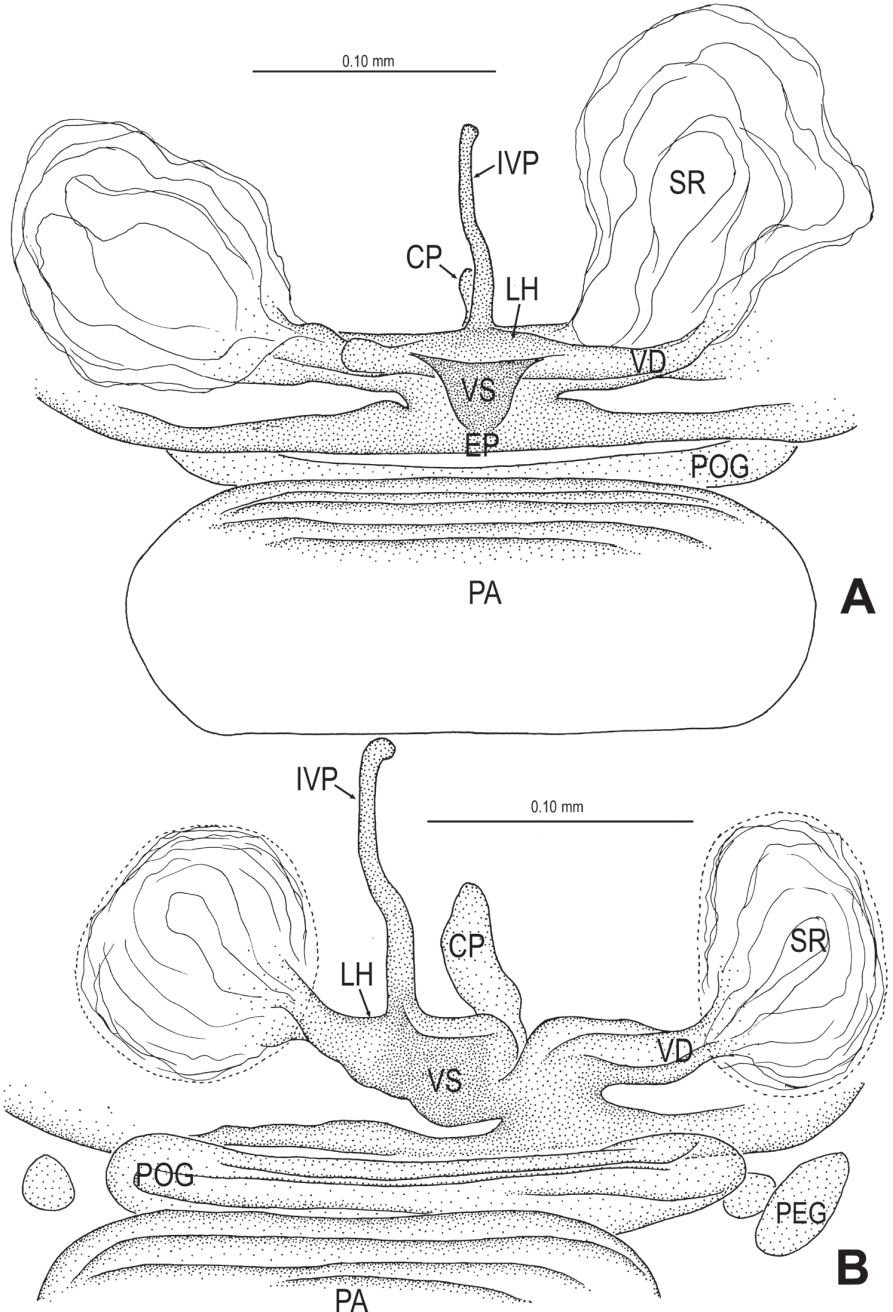
*Tetrablemma menglaensis* sp. n., female paratype. **A, B** Cleared vulva (KOH-treated) **A** dorsal view **B** dorsal-lateral view. Abbrs.: **CP** central process; **EP** epigynal pit; **IVP** inner vulval plate; **LH** lateral horn; **PA** preanal plate; **POG** postgenital plate; **SR** seminal receptaculum; **VD** vulval duct; **VS** vulval stem.

**Figure 22.**
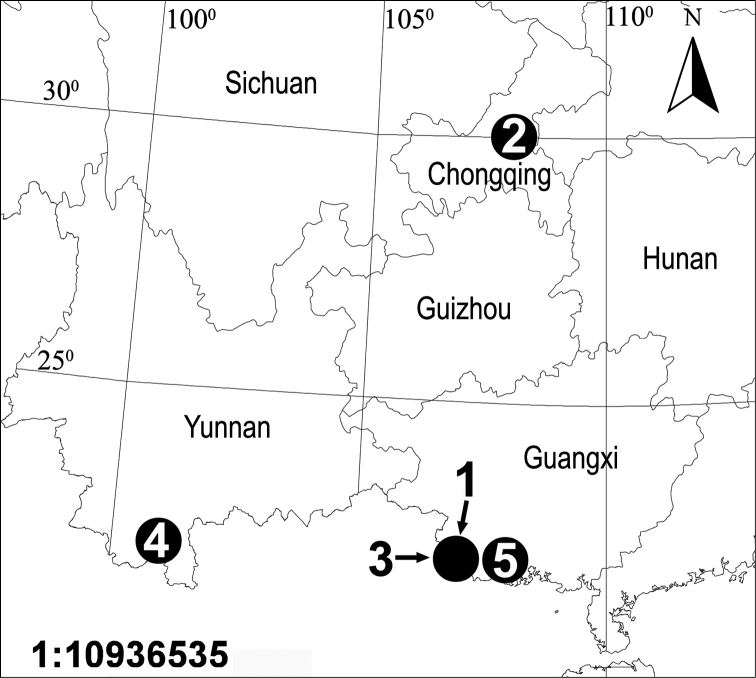
Distribution records of five new tetrablemmid species from China. **1** *Singaporemma banxiaoensis* sp. n. **2** *Singaporemma wulongensis* sp. n. **3** *Sinamma oxycera* gen. n. & sp. n. **4** *Tetrablemma menglaensis* sp. n. **5** *Tetrablemma ziyaoensis* sp. n.

## Supplementary Material

XML Treatment for Sinamma

XML Treatment for Sinamma oxycera

XML Treatment for Singaporemma

XML Treatment for Singaporemma banxiaoensis

XML Treatment for Singaporemma wulongensis

XML Treatment for Tetrablemma

XML Treatment for Tetrablemma menglaensis

XML Treatment for Tetrablemma ziyaoensis
